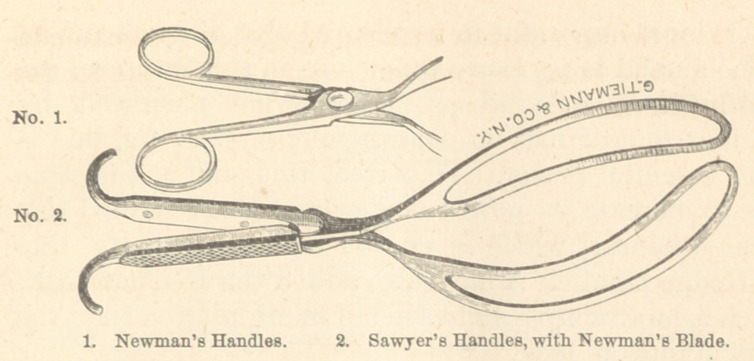# A New Obstetric Forceps

**Published:** 1876-01

**Authors:** Edw. Warren Sawyer

**Affiliations:** Lecturer on Obstetrics, Rush Medical College, Chicago


### A NEW OBSTETRIC FORCEPS.

Extract from a paper on the Second Stage of Labor; read before the Chicago Society of Physicians and Surgeons, Oct. 25, 1875.

By EDW. WARREN SAWYER, M.D.,
Lecturer on Obstetrics, Rush Medical College, Chicago.

Till this time, I have scarcely used another instrument than the long, double-curved forceps of Simpson: preferring a skilled acquaintance with a single instrument to an imperfect knowledge of many; and because the long forceps are capable of almost all applications. I have always wished, however, for a less bulky instrument, which could be applied to the frequent cases where it is wished to disengage the head from the inferior strait, or to lift it from the perineum; but the ordinary short forceps are not more graceful, and scarcely more easily applied, than the long. Recently, my friend, Dr. Wm. H. Newman, of Denver, Colorado, has given us an instrument which, I must say, does seem to fill the want.

This graceful little instrument is, truly, the baby forceps; it is so small it can be easily carried in the pocket, its entire length being but nine and one-half inches; the length of the handle to the lock is three inches; the length of shank from lock to curve is one inch; the blade is uniformly one inch wide and one-sixteenth of an inch in thickness; it has a pelvic and head curve; the chord of the arc represented by the head curve is six inches in length; the instrument weighs two and three-fourths ounces. The short scissors-handle and the delicate structure of the instrument, give it a less formidable appearance than that of any other forceps with which I am acquainted.

No. 1.

No. 2.

1. Newman's Handles. 2. Sawyer's Handles, with Newman's Blade.

With the thumb and a single finger, which is all that is required in its use, this instrument possesses no compressing power, other than that the uterus invokes through its blades; so that it may be pronounced a safe instrument. I think it no exaggeration to say that it will be impossible with this forceps to do harm, either to the mother or fœtus. No operation in obstetrics, not even uncomplicated catheterization, is as simple as the application of this instrument, which can almost be done without the knowledge of the woman. Obviously its use is limited to those cases I have already indicated, viz., when the head rests upon the perineum, or, at most, is in the lower strait.

No one has yet used the instrument as extensively as the inventor, from whose recent letter I extract the following: "My forceps surpass my own expectation. I was a little disappointed in the first two or three cases—I was awkward with the button lock; but now that I have learned how and when to use them, I find them all right. * * * * I have had about twelve cases of labor since I received the forceps, and have used them in nine of these cases. I do not now sit by a slow case, giving ergot, encouraging the patient and assuring the friends around, that all will be right after a while, but I apply the forceps and deliver in from ten to thirty minutes. I am sure that, in at least two of these cases, two or three hours would have been required for the completion of labor."

It is most important to be assured that the instrument in one's hand is perfectly safe; but, in this instance, the usefulness of the forceps is somewhat narrowed by the fact that it has no compressing power; for this is often essential power. To correct this, and at the same time to increase its power as a tractor, I have added this serrated handle, which is curved at the extremity, like the Hodge handle. I have also added the Denman lock, which is more simple and easier of manipulation than the English button lock.

The execution of these changes I have entrusted to the Messrs. Tiemann & Co., of New York, who have accomplished the result with their usual skill.

---

*The danger of keeping flowers and fruits in sleeping rooms* is anew illustrated by the following instances, reported by Dr. Breitter, (*Wiener Med. Presse*, 43, 1875): A gentleman had the unhappy idea of making of the branches of an oleander some sort of an alcove in which to sleep; next morning he was found dead. A grocer and his clerk went to sleep in a room in which three boxes of oranges stood, and they were dead by the next morning. A clerk in a store who was to watch at night, laid down with a bag of sassafras under his head, he likewise was found dead in the morning. Another gentlemen having some hyacinths in his room, got the most violent headache and felt so drowsy that he could scarcely restrain from sleeping. He at once put the flowers out of the room, opened the windows, and soon after felt easier.

For the Removal of Comedones from the Face.—Gutceit recommends the washing and rubbing of the skin with diluted liq. ammoniæ, one teaspoonful to a wine glassful of water.—*Memorabilien*, xx, 7.

## Figures and Tables

**Figure f1:**